# Effect of prebiotics, probiotics, synbiotics on depression: results from a meta-analysis

**DOI:** 10.1186/s12888-023-04963-x

**Published:** 2023-06-29

**Authors:** Qin Zhang, Bing Chen, Jinghui Zhang, Jingyi Dong, Jianglin Ma, Yuyan Zhang, Kangyu Jin, Jing Lu

**Affiliations:** 1 First Clinical Medical College, Zhejiang Chinese Medical University, Hangzhou, China; 2 Department of Psychiatry, The First Affiliated Hospital, Zhejiang University School of Medicine, Hangzhou, China; 3 School of Life Sciences, Zhejiang Chinese Medical University, Hangzhou, China; 4 Department of Pediatrics, The First Affiliated Hospital, Zhejiang University School of Medicine, Hangzhou, China; 5 Key Laboratory of Mental Disorder Management in Zhejiang Province, Hangzhou, China

**Keywords:** Probiotics, Prebiotics, Synbiotics, Depression, Gut microbiota

## Abstract

Accumulating studies have shown the effects of gut microbiota management tools in improving depression. We conducted a meta-analysis to evaluate the effects of prebiotics, probiotics, and synbiotics on patients with depression. We searched six databases up to July 2022. In total, 13 randomized controlled trials (RCTs) with 786 participants were included. The overall results demonstrated that patients who received prebiotics, probiotics or synbiotics had significantly improved symptoms of depression compared with those in the placebo group. However, subgroup analysis only confirmed the significant antidepressant effects of agents that contained probiotics. In addition, patients with mild or moderate depression could both benefit from the treatment. Studies with a lower proportion of females reported stronger effects for alleviating depressive symptoms. In conclusion, agents that manipulate gut microbiota might improve mild-to-moderate depression. It is necessary to further investigate the benefits of prebiotic, probiotic and synbiotic treatments relative to antidepressants and follow up with individuals over a longer time before these therapies are implemented in clinical practice.

**Supplementary Information:**

The online version contains supplementary material available at 10.1186/s12888-023-04963-x.

## Introduction

Major depressive disorder (MDD) is a mood disorder that impairs psychosocial function and quality of life. The crude prevalence of depression or depressive symptoms was 27.2%, according to the data extracted from 183 studies in 43 countries [1]. Depression causes more ‘years lost’ to disability than any other condition and is ranked by the World Health Organization (WHO) as the third leading cause of global disease burden of disease [2, 3]; by 2030, depression is projected to reach first place. The initial therapeutic modality for depression is pharmacotherapy with or without psychotherapy and other somatic therapies. A previous meta-analysis assessed the efficacy of 21 antidepressant drugs in treating MDD and found that all antidepressants were more efficacious than placebo [4]. However, approximately 50% of patients with MDD insufficiently respond to antidepressant drugs [5]. In addition, a study analyzed 8262 patients with MDD from 28 placebo-controlled SSRI trials and found that patients with mild or moderate MDD showed a less pronounced response to treatment than those with severe MDD [6]. The side effects of antidepressants can include sexual dysfunction, weight gain, and daytime sleepiness, thus leading to the high discontinuation rate of antidepressant treatment [7, 8]. These problems necessitate the development of adjunctive treatments such as gut microbiota management tools, behavioral activation, and somatic therapies.

Gut microbiota management tools encompass prebiotics, probiotics, and synbiotics. Probiotics are live microorganisms that provide a health benefit when consumed in adequate amounts [9]. Prebiotics refer to a substrate selectively utilized substrate by host microorganisms conferring a health benefit [10]. In addition, synbiotics are a mixture of prebiotics and probiotics [11]. Burgeoning researches has explored the therapeutic effects of these gut microbiota management tools in the recent years. These agents modulate the internal microbiota and its function and then exert an impact on the central nervous system (CNS) via neural, neuroendocrine, neuroimmune, and humoral links [12]. Therefore, prebiotics, probiotics, and synbiotics reveal a novel way to treat psychiatric disorders such as depression through the microbiota-gut-brain axis [13]. It has been proved in animal models that probiotics or prebiotics are capable of alleviating depressive-like behaviors, but data from clinical studies are still scarce and unconvincing [14–17]. Therefore, it is necessary to perform a meta-analysis to evaluate the effect of prebiotics, probiotics and synbiotics on patients with depression, which also provides a reference for further research.

There have also been several systematic reviews and meta-analyses of the relevant topic due to the rapidly growing interest in this realm [18–20]. In comparison with them, our study included more homogeneous subjects (excluding comorbid depression and healthy individuals), more comprehensive interventions including prebiotics, probiotics and synbiotics, and more diverse outcome indicators to assess the effectiveness. More importantly, we included several recent studies that were not included in the previous analyses. In general, the study aims to figure out whether gut microbiota management tools could exert significant effects on depression. The primary objective of this study is to evaluate the effectiveness of prebiotics, probiotics and synbiotics in alleviating depressive symptoms. The secondary objectives are to explore influential factors associated with their effectiveness, and summarize alterations in gut microbiota depicted by different indices and changes in depression-related biochemical indicators.

## Method

### Search strategy

This meta-analysis was performed in accordance with Preferred Reporting Items for Systematic Reviews and Meta-analyses (PRISMA) principles [21]. The study protocol was registered at PROSPERO (registration ID: CRD42022373150). Two reviewers independently searched six databases, including PubMed, Cochrane Library, Embase, Medline, Web of Science and PsycINFO. The following search query was formed with Medical Subject Heading (MeSH) terms and entry terms as search filters: (“Depression”[Mesh] OR “Depressive Disorder”[Mesh] OR “unipolar depression” OR “mental depression”) AND (“Prebiotics”[Mesh] OR “Probiotics”[Mesh] OR “Synbiotics”[Mesh]). A more specific strategy is provided in Supplementary Table 1. The references of similar systematic reviews and meta-analyses focused on prebiotics, probiotics and psychiatric disorders were also searched. The retrieval was not limited by language and included articles up to July 2022.

### Study selection

Two reviewers independently evaluated the eligibility of retrieved studies, and any discrepancies were submitted to the corresponding author for resolution. Titles and abstracts of all articles were initially screened, and then full texts were carefully assessed according to the inclusion and exclusion criteria.

Studies were included if they met the following criteria: (1) RCTs in humans focused on the effects of prebiotics, probiotics, and synbiotics on depression; (2) patients with the clinical diagnosis of depression based on DSM-IV/V, ICD-10 or validated depression rating pools; (3) probiotics and/or prebiotics and/or synbiotics were used as treatment; (4) the control group received undistinguished placebo; and (5) rating scales for depression and/or gastrointestinal microbiota were assessed before and after the intervention;

The study exclusion criteria were as follows: (1) patients with a comorbidity of major psychiatric or physical diseases (e.g., bipolar disorder or irritable bowel syndrome [IBS]), or healthy participants; (2) the use of prebiotics and/or prebiotics and/or synbiotics was self-reported instead of prescriptions from doctors; (3) the data of intestinal microbiota and/or depressive rating scales were missed, incomplete or unavailable; (4) reviews, meta-analyses, observational studies, case reports, studies on animals or cell lines, comments, abstracts from conferences and unpublished clinical trials; and (5) full texts were unavailable or main contents were duplicated.

### Data extraction

Two reviewers independently extracted the data from the included articles, and any conflicts were resolved by discussion with the corresponding author. The following data were extracted: (1) basic information of studies including first author, publication year, country, and study design; (2) population characteristics including clinical diagnosis of depression, diagnostic criteria, duration of depression, age, sex, body mass index (BMI), education; (3) intervention characteristics including the types of intervention (prebiotics, probiotics or synbiotics), probiotic strains, dosage, intervention duration, usage of antidepressant drugs; (4) the change in rating scales for depression, depression-related biomedical indicators, microbiome taxa, α and β diversity (α diversity represents the richness and evenness of the microbial community in individual samples, while β diversity evaluates interindividual diversity that assesses dissimilarity of microbial communities compared with the other samples analyzed).

### Quality assessment

The risk of bias in the included studies was evaluated by two independent reviewers using Cochrane Collaboration’s Risk of bias Tool 2 [22]. The judgement of overall bias is based on the evaluation of 5 domains, including the randomization process, deviations from intended interventions, missing outcome data, measurement of the outcome, and selection of the reported result. The quality of evidence was assessed by the Grading of Recommendation, Assessment, Development, and Evaluation (GRADE) scale (https://gdt.gradepro.org).

### Statistical analysis

We utilized the change in depressive symptom score, microbiota indices and inflammatory indicators to make comparisons between interventional and placebo groups (change value = post-intervention—baseline). The data transformation was based on the Cochrane Handbook for Systematic Reviews of Interventions version 6.3 (http://www.handbook.cochrane.org). If one study contained more than one eligible intervention group, in order to overcome the unit-of-analysis error, the control groups were divided into smaller sample sizes with unchanged mean and standard deviation (*SD*). The standardized mean difference (*SMD*) was calculated to pool the continuous results. 95% confidence interval (*CI*) and two-sided *P* values were used for each outcome. Heterogeneity among different studies was examined using the *I*^*2*^ statistic and Q-test. A fixed-effects model was selected in cases of low heterogeneity (*P* ≥ 0.05 or *I*^*2*^ < 50%); otherwise, a random-effects model was used because it attempted to generalize findings beyond the included studies by assuming that the selected studies are random samples from a larger population [23–25]. Subgroup analysis was conducted to explore the heterogeneity and moderators that affected the outcomes. Overall studies were stratified based on (1) the percentage of females included in studies; (2) different disease severity (moderate or mild depression); (3) whether prebiotics or agents containing probiotics (i.e., probiotics and synbiotics) were used for intervention; (4) single or multiple strains of probiotics; (5) whether prebiotics, probiotics, and synbiotics were used as an adjunctive therapy; (6) the intervention duration; (7) different assessment tools of depression; and (8) whether the assessment of depression was performed at the end of the intervention or after a follow-up period. Multiple meta-regression was a quantified analysis to further interpret the heterogeneity and evaluate effects of different factors on the outcomes. Participant characteristics and several factors in the subgroup analysis were included as explanatory covariates. A sensitivity analysis was conducted to examine the robustness of the results. Egger’s test, Begg’s test, and funnel plots were used to examine potential publication bias. If there was a possible publication bias, the “trim and fill” method was utilized [26, 27]. This meta-analysis was performed using Stata/SE 15.1 (Stata Corporation, TX, USA).

## Results

### Study selection and study characteristics

**Fig. 1:**
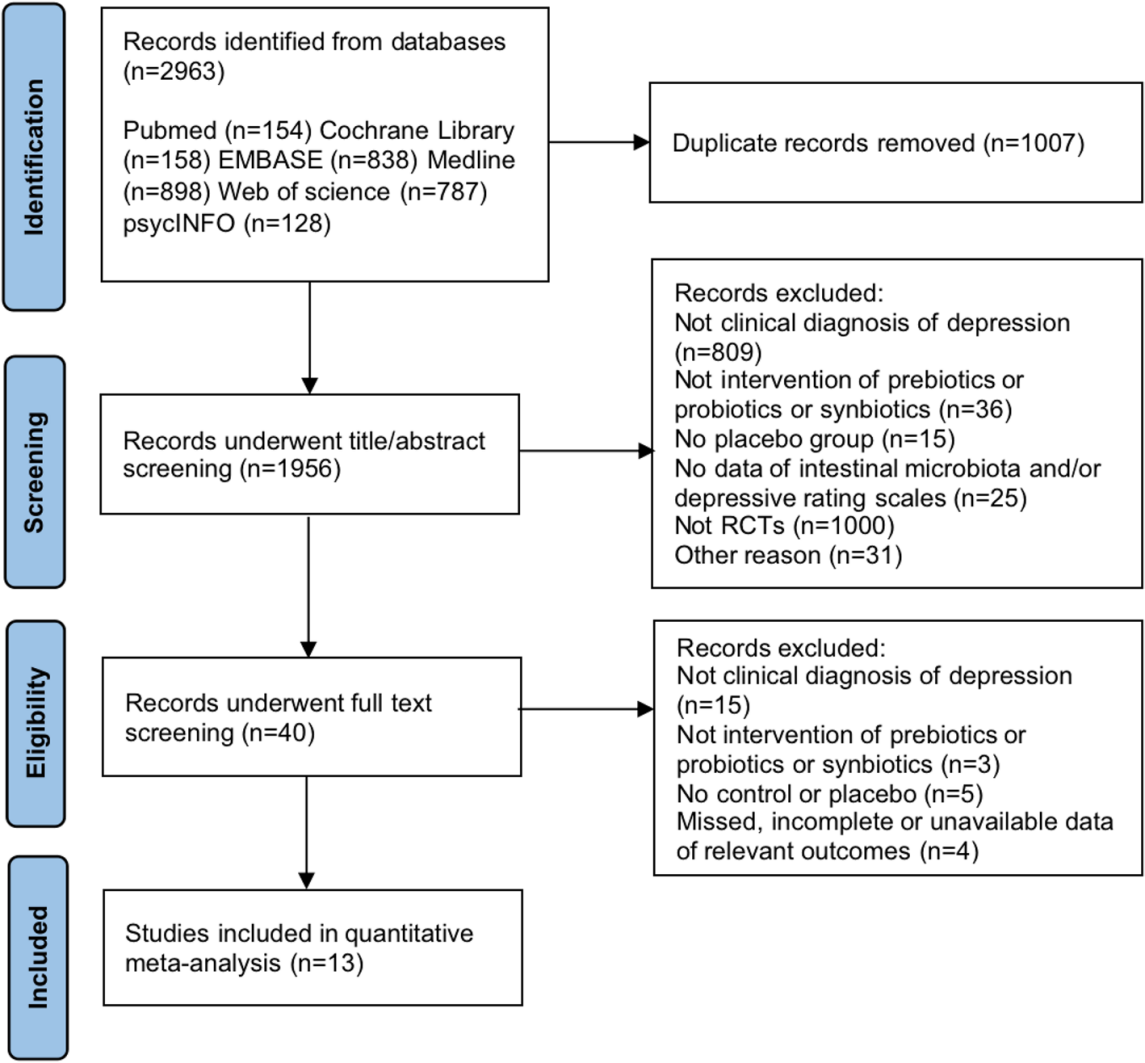
PRISMA flow diagram of the study

The study flow diagram is shown in Fig. 1. A total of 2963 records were retrieved from six databases, and 1007 duplicates were removed. After screening the titles, abstracts and full texts, 13 studies were finally included in this meta-analysis [28–41]. All studies were published online between 2016 and 2022. Overall, 786 participants were allocated to the intervention group (*n* = 427) and the placebo group (*n* = 359). Most participants were female (a percentage ≥ 50% in all studies). The mean age of each study ranged from 34.5 to 53.0 years. For interventions, 9 studies compared probiotics and placebo, 1 study compared prebiotics and placebo, and 1 study compared synbiotics and placebo. Heidarzadeh-Rad et al. and Kazemi et al. performed three-armed comparisons between prebiotics, probiotics and placebo. These two studies initially recruited the same population, but adopted PP (per-protocol) or ITT (intention-to-treat) analysis, respectively. The intervention duration ranged from 3 to 24 weeks. The outcomes of scales that measured depressive symptoms varied among different researchers. The scales included the Hamilton Depression Rating Scale (HDRS) (*n* = 8), Beck Depression Inventory (BDI) (*n* = 5), Montgomery Asberg Depression Rating Scale (MADRS) (*n* = 3), and Edinburgh Postnatal Depression Scale (EPDS) (*n* = 1). Detailed characteristics of the study are shown in Table 1.

**Table 1:** Basic characteristics of the included studies

Study	Country	Population (Diagnosis criteria)	Age Mean (*SD*)	Sex (%female)	Intervention	Control	Intervention duration	Follow-up period	Outcome measures
Akkasheh, G., et al. (2016) [28]	Iran	MDD (DSM-IV and HDRS-17 ≥ 15)	Pro: 38.3 (12.1) Ct: 36.2 (8.2)	Pro:85.0 Ct: 85.0	Probiotics (*n* = 20)	Placebo (*n* = 20)	8 weeks	No	BDI
Browne, P.D., et al. (2021) [29]	Netherlands	Pregnant woman with depressive symptoms (EPDS ≥ 10)	Pro: 29.7 (3.9) Ct: 31.7 (4.0)	Pro: 100 Ct: 100	Probiotics (*n* = 20)	Placebo (*n* = 20)	8 weeks	4 weeks post-partum	EPDS
Ghorbani, Z., et al. (2018) [30]	Iran	MDD (DSM-V and HDRS-17 of 17–23)	Syn: 34.5 (4.0) Ct: 35.5 (5.27)	Syn: 70.0 Ct: 70.0	Synbiotics + fluoxetine (*n* = 20)	Placebo + fluoxetine (*n* = 20)	6 weeks	No	HDRS
Heidarzadeh-Rad, N., et al. (2020) [31]	Iran	MDD (clinical diagnosis of the psychiatrist)	Pro: 37.8 (7.9) Pre: 36.6 (8.4) Ct: 36.0 (8.5)	Pro: 71.4 Pre: 80.0 Ct: 60.0	Probiotics (*n* = 28) or prebiotics (*n* = 25) + antidepressant medications	Placebo + antidepressant medications (*n* = 25)	8 weeks	No	BDI
Huang, W., et al. (2019) [32]	China	MDD (ICD-10 + CCMD-3)	/	/	Probiotics + electroacupuncture (*n* = 56)	Trimebutine maleate + meptintin (*n* = 48)	3 weeks	No	HDRS
Kazemi, A., et al. (2019) [33, 34]	Iran	MDD (Clinical diagnosis of the psychiatry clinic)	Pro: 36.2 (7.9) Pre: 37.4 (8.0) Ct: 36.0 (8.5)	Pro: 71.1 Pre: 75.0 Ct: 66.7	Probiotics (*n* = 38) Prebiotics (*n* = 36)	Placebo (*n* = 36)	8 weeks	No	BDI
Reininghaus, E.Z., et al. (2020) [35]	Austria	Depressive episode (Interview M.I.N.I. by a psychiatric)	Pro: 43.0 (14.3) Ct: 40.1 (11.5)	Pro: 71.4 Ct: 81.8	Probiotic + pharmaceuticals (*n* = 28)	Placebo + pharmaceuticals (*n* = 30)	4 weeks	No	HDRS BDI
Romijn, A.R., et al. (2017) [36]	New zealand	QIDS-SR16 ≥ 11 or DASS-42 ≥ 14	Pro: 35.8 (14.0) Ct: 35.1(14.5)	Pro: 80.0 Ct: 76.9	Probiotics (*n* = 40)	Placebo (*n* = 39)	8 weeks	No	MADRS QIDS-SR16 DASS-42
Rudzki, L., et al. (2019) [37]	Poland	MDD (DSM-IV)	Pro:39.1(10.0) Ct: 38.9 (12.0)	Pro: 76.7 Ct: 66.7	Probiotics + SSRI (*n* = 30)	Placebo + SSRI (*n* = 30)	8 weeks	No	HDRS SCL-90 PSS-10
Schaub, A.C., et al. (2022) [38]	Switzerland	Depressive episode (ICD-10 or HDRS > 7)	Pro: 39.2 (11.5) Ct: 38.0 (10.2)	Pro: 73.7 Ct: 50.0	Probiotics (*n* = 19)	Placebo (*n* = 24)	4 weeks	4 weeks after the intervention	HDRS BDI
Tarutani, S., et al. (2022) [39]	Japan	MDD (ICD-10 and CGI < 5 and ≥ 2)	Pre: 54.3 (10.0) Ct: 53.4 (11.3)	Pre: 88.9 Ct: 81.8	Prebiotics (*n* = 9)	Placebo (*n* = 11)	24 weeks	No	MADRS
Tian, P., et al. (2022) [40]	China	MDD (HDRS > 24)	Pro: 51.3(16.1) Ct: 48.2(14.0)	Pro: 70.0 Ct: 64.0	Probiotics + antidepressant medications (*n* = 20)	Placebo + antidepressant medications (*n* = 25)	4 weeks	No	HDRS MADRS BPRS
Zhang, X., et al. (2021) [41]	China	Depression (DSM-5)	Pro: 45.8 (12.3) Ct: 49.7 (9.6)	Pro: 63.2 Ct: 64.5	Probiotics + antidepressant medications (*n* = 38)	Placebo + antidepressant medications (*n* = 31)	9 weeks	No	HDRS BDI

*BDI* Beck Depression Inventory, *BPRS* Brief Psychiatric Rating Scale, *CCMD* Chinese Classification of Mental Disorders, *CGI* Clinical Global Impression, *Ct* Control, *DASS-42* Depression, Anxiety and Stress Scale, *DSM* Diagnostic and Statistical Manual of Mental Disorders, *EPDS* Edinburgh Postnatal Depression Scale, *HDRS* Hamilton Depression Rating Scale, *ICD* International Statistical Classification of Diseases and Health Related Problems, *MADRS* Montgomery Asberg Depression Rating Scale, *MDD* Major depression disease, *Pre* Prebiotics, *Pro* Probiotics, *QIDS-SR16* Quick Inventory of Depressive Symptomatology, *SSRI* Selective serotonin reuptake inhibitors, *SCL-90* Symptom Checklist, *PSS-10* Perceived Stress Scale, *Syn* Synbiotics

### Effect of prebiotics, probiotics or synbiotics on depression symptoms

**Fig. 2:**
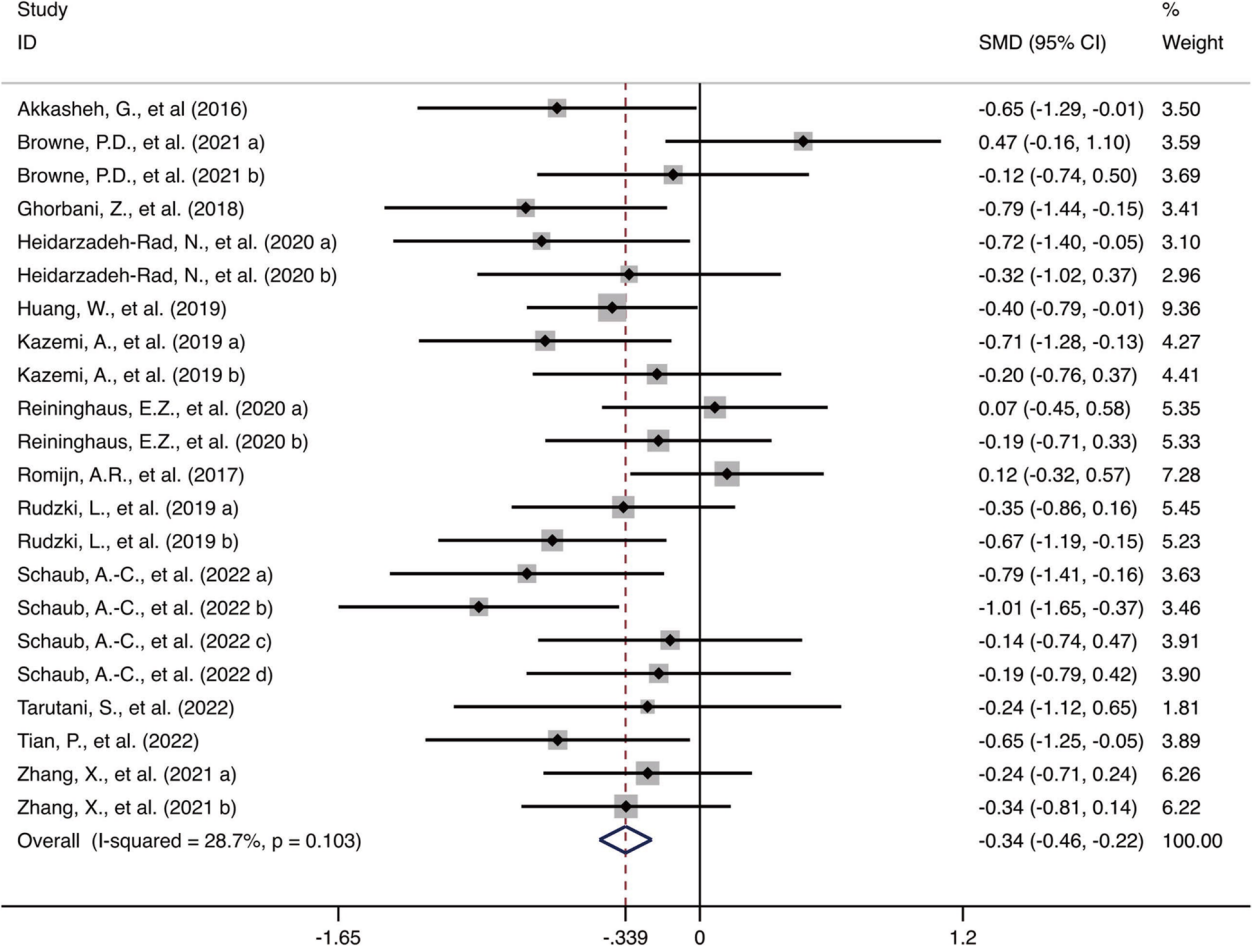
Forest plot of the studies investigating the effect of prebiotics, probiotics and synbiotics in improving depressive symptoms compared with the placebo

Thirteen studies with 22 treatment and control groups were pooled to evaluate the efficacy of prebiotics, probiotics and synbiotics. In the studies in which depressive symptoms were assessed by different rating scales (HDRS or BDI) or at the different time points (during the intervention, the end of intervention or follow-up), multiple groups of data were all included in the meta-analysis [29, 35, 37, 38, 41]. For three-armed studies consisting of prebiotics, probiotics and placebo groups, we divided the data into two sets (probiotics vs. placebo, prebiotics vs. placebo) in the meta-analysis [31, 33, 34]. Figure 2 shows that patients who received prebiotic, probiotic or synbiotic treatment had significant improvement in depression compared with those in the placebo group (*SMD* = -0.34 [-0.45, -0.22], *P* < 0.001). Moreover, the heterogeneity of the outcomes was low (*I*^*2*^ = 28.7%, *P* = 0.103).

### The results of the subgroup analysis

**Fig. 3:**
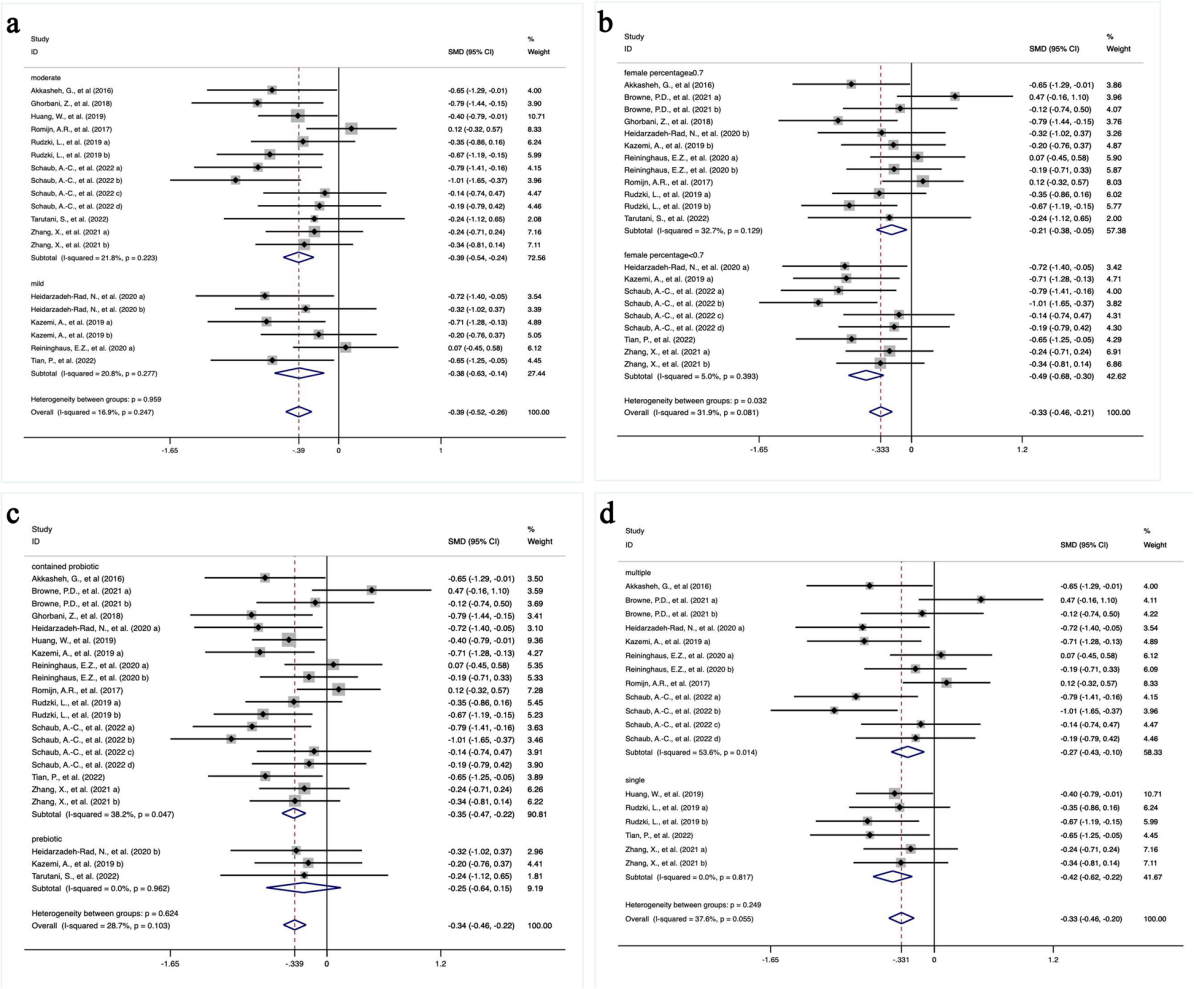
The outcomes of subgroup analysis. **a** based on the severity of depression, **b** based on whether the percentage of females in the study population was more or less than 70%, **c** based on different types of interventional agents, including prebiotics or agents containing probiotics (i.e., probiotics and synbiotics), **d** based on single or multiple strains of probiotics

The first subgroup analysis examined the influence of depression severity on the primary outcomes (Fig. 3a). Probiotics, prebiotics and synbiotics were significantly superior to placebo in improving depressive symptoms in patients with both mild and moderate depression (mild: *SMD* = -0.38 [-0.63, -0.14], *P* = 0.002; moderate: *SMD* = -0.39 [-0.54, -0.24], *P* < 0.001). In addition, heterogeneity within subgroups and between subgroups was insignificant (mild: *I*^*2*^ = 20.8%, *P* = 0.277; moderate: *I*^*2*^ = 21.8%, *P* = 0.223; heterogeneity between groups: *P* = 0.959).

Most of the participants in the included studies were female. The percentage of females might induce different outcomes between studies (Fig. 3b). The intervention could significantly alleviate depressive symptoms regardless of the percentage of females, with low heterogeneity within either subgroup (female rate ≥ 0.7: *I*^*2*^ = 32.7%, *P* = 0.129; female rate < 0.7: *I*^*2*^ = 5.0%, *P* = 0.393). More importantly, the heterogeneity between groups was also significant (*P* = 0.032). Studies containing a lower percentage of females (< 70%) had a larger reduction in depressive symptom scores with an *SMD* of -0.49 (*95% CI* [-0.68, -0.30], *P* < 0.001), compared with those consisting of more than 70% females (*SMD* = -0.21 [-0.38, -0.05], *P* = 0.011).

The third subgroup analysis was based on different types of interventional agents, including prebiotics or agents containing probiotics (i.e., probiotics and synbiotics) (Fig. 3c). The pooled effects of agents containing probiotics on depression were significant (*SMD* = -0.35[-0.47, -0.22], *P* < 0.001), accompanied by slightly increased heterogeneity (*I*^*2*^ = 38.2%, *P* = 0.047). However, in three studies with prebiotic intervention, the reduction in depressive symptom scores showed no significant difference from the placebo group (*SMD* = -0.25[-0.64,0.15], *P* = 0.221). The heterogeneity of the prebiotic subgroup was nonsignificant (*I*^*2*^ = 28.7%, *P* = 0.103). In addition, the heterogeneity between those two groups was not significant (*P* = 0.624).

Amongst studies applying probiotics as the intervention, 4 studies used a single strain, and the remaining 7 studies used multiple strains. The depressive symptom scores were reduced significantly, regardless of whether single or multiple strains were applied (multiple: *SMD* = -0.27 [-0.43, -0.10], *P* = 0.002; single: *SMD* = -0.42[-0.62, -0.22], *P* < 0.001). Additionally, no significant heterogeneity between groups was observed (*P* = 0.249), with significant heterogeneity within the subgroup of multiple strains (*I*^*2*^ = 53.6%, *P* = 0.014), and absent heterogeneity within the subset of single strain (*I*^*2*^ = 0.0%, *P* = 0.817) (Fig. 3d).

**Fig. 4:**
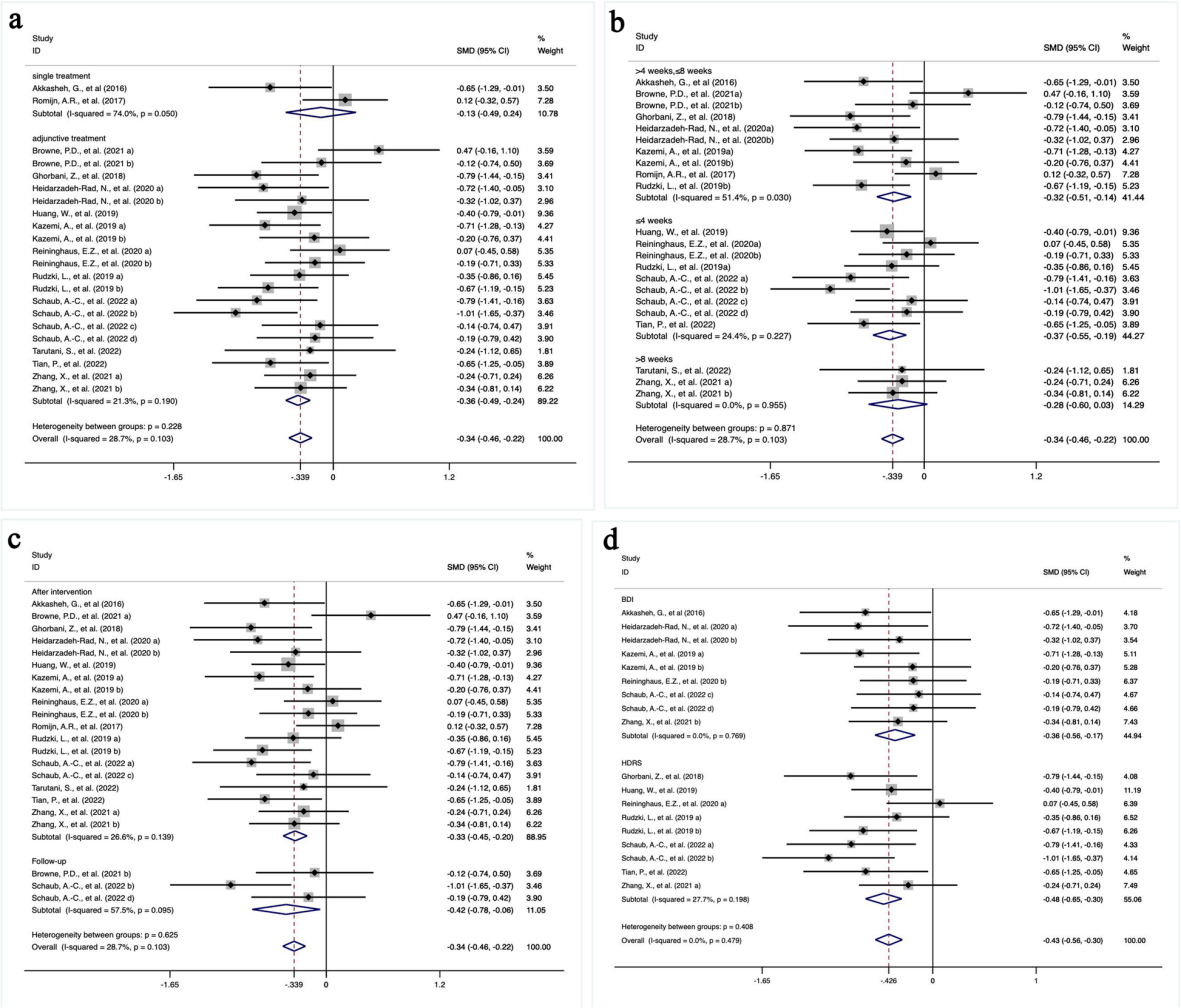
**a** based on whether prebiotics, probiotics and synbiotics were used as adjunctive therapy, **b** based on different intervention duration (i.e., ≤ 4 weeks, 4 to 8 weeks and > 8 weeks), **c** based on whether the assessment of depression was performed at the end of the intervention or after a follow-up period, **d** based on the different assessment tools of depressive symptom (HDRS or BDI)

As presented in Fig. 4a, the fifth subgroup analysis divided studies by whether prebiotics, probiotics and synbiotics were used as a single or adjunctive treatment. Adjunctive treatment was observed to improve depression compared with placebo, with an *SMD* of -0.36 (*95% CI* [-0.49, -0.24], *P* < 0.001) and nonsignificant heterogeneity (*I*^*2*^ = 21.3%, *P* = 0.190). The therapeutic effects of a single treatment were not significantly different from the placebo (*SMD* = -0.13[-0.49, 0.24], *P* = 0.490) along with high heterogeneity within the subgroup (*I*^*2*^ = 74.0%, *P* = 0.050). The heterogeneity between the two groups was insignificant (*P* = 0.228).

According to different lengths of treatment, studies were stratified into three subgroups including ≤ 4 weeks, 4 to 8 weeks and > 8 weeks (Fig. 4b). Subgroups with a treatment duration of fewer than 8 weeks had significant beneficial effects on depressive symptoms (≤ 4 weeks: *SMD* = -0.37[-0.55, -0.19], *P* < 0.001; 4 to 8 weeks: *SMD* = -0.32[-0.51, -0.14], *P* = 0.001). Patients who received more than 8 weeks of therapy failed to have a significant *SMD* of -0.28 (*95% CI* [-0.60, 0.03], *P* = 0.080). However, those three groups did not have significant heterogeneity (*P* = 0.871). Heterogeneity was evident in the subgroup of 4 to 8 weeks treatment duration (*I*^*2*^ = 51.4%, *P* = 0.030), while nonsignificant heterogeneity was observed in the other subgroups (≤ 4 weeks: *I*^*2*^ = 24.4%, *P* = 0.227; > 8 weeks: *I*^*2*^ = 0.0%, *P* = 0.955).

The assessment of depressive symptom scores whether at the end of the intervention or after a follow-up period might affect the outcomes. As demonstrated in Fig. 4c, probiotics, prebiotics and synbiotics exerted significant therapeutic effects on depression either evaluated after the intervention or after a follow-up period (after intervention: *SMD* = -0.33[-0.45, -0.20], *P* < 0.001; after follow-up: *SMD* = -0.42[-0.78, -0.06], *P* = 0.021). Additionally, the heterogeneity within each subgroup and between subgroups was nonsignificant (after intervention: *I*^*2*^ = 26.6%, *P* < 0.139; after follow-up: *I*^*2*^ = 57.5%, *P* = 0.095; heterogeneity between subgroups: *P* = 0.625).

The last subgroup analysis was based on different depressive rating scales. Figure 4d shows that whether assessed by HDRS or BDI, the overall efficacy of prebiotics, probiotics and synbiotics in improving depressive symptoms was more significant than placebo (HDRS: *SMD* = -0.48 [-0.65, -0.30], *P* < 0.01; BDI: *SMD* = -0.36[-0.56, -0.17], *P* < 0.01). The heterogeneity of each subgroup and between subgroups was nonsignificant (HDRS: *I*^*2*^ = 27.7%, *P* = 0.198; BDI: *I*^*2*^ = 0.0%, *P* = 0.769; heterogeneity between subgroups: *P* = 0.408).

### The result of meta-regression

Moderators were put in multivariant meta-regression analysis to demonstrate their influence on primary outcomes. Table 2 shows that the percentage of females significantly affected the efficacy of the intervention. A more considerable reduction in depressive symptoms was observed in the studies that contained a lower percentage of females (*coefficient* = 1.925, *P* = 0.026). Additionally, age (*P* = 0.152), treatment duration (≤ 4 weeks vs. 4 to 8 weeks, *P* = 0.076; > 8 weeks vs. 4 to 8 weeks, *P* = 0.064), intervention agent (*P* = 0.643), intervention type (*P* = 0.423) and evaluation timepoint (*P* = 0.292) showed no significant influence on the treatment effects.

**Table 2:** Meta-regression of the efficacy of prebiotics, probiotics and synbiotics on depression

Covariates	*Coefficient*	*Standard error*	*t*	*P*	*95%CI*	
Treatment duration (reference = > 4 weeks, ≤ 8 weeks)						
≤ 4 weeks	0.494	0.256	1.93	0.076	-0.059	1.046
> 8 weeks	0.849	0.419	2.03	0.064	-0.056	1.753
Age	-0.043	0.028	-1.52	0.152	-0.104	0.018
Percentage of females	1.925	0.766	2.51	0.026^*^	0.271	3.580
Intervention agent (Probiotics or non-probiotics)	-0.109	0.231	-0.47	0.643	-0.608	0.389
Intervention type (Single or adjunctive intervention)	-0.211	0.256	-0.83	0.423	-0.764	0.341
Evaluation timepoint (After intervention or follow-up)	-0.264	0.241	-1.10	0.292	-0.784	0.256

*CI* Confidence interval

^*^P < 0.05

### The change in α diversity, β diversity, and the abundance of specific microbiome

**Fig. 5:**
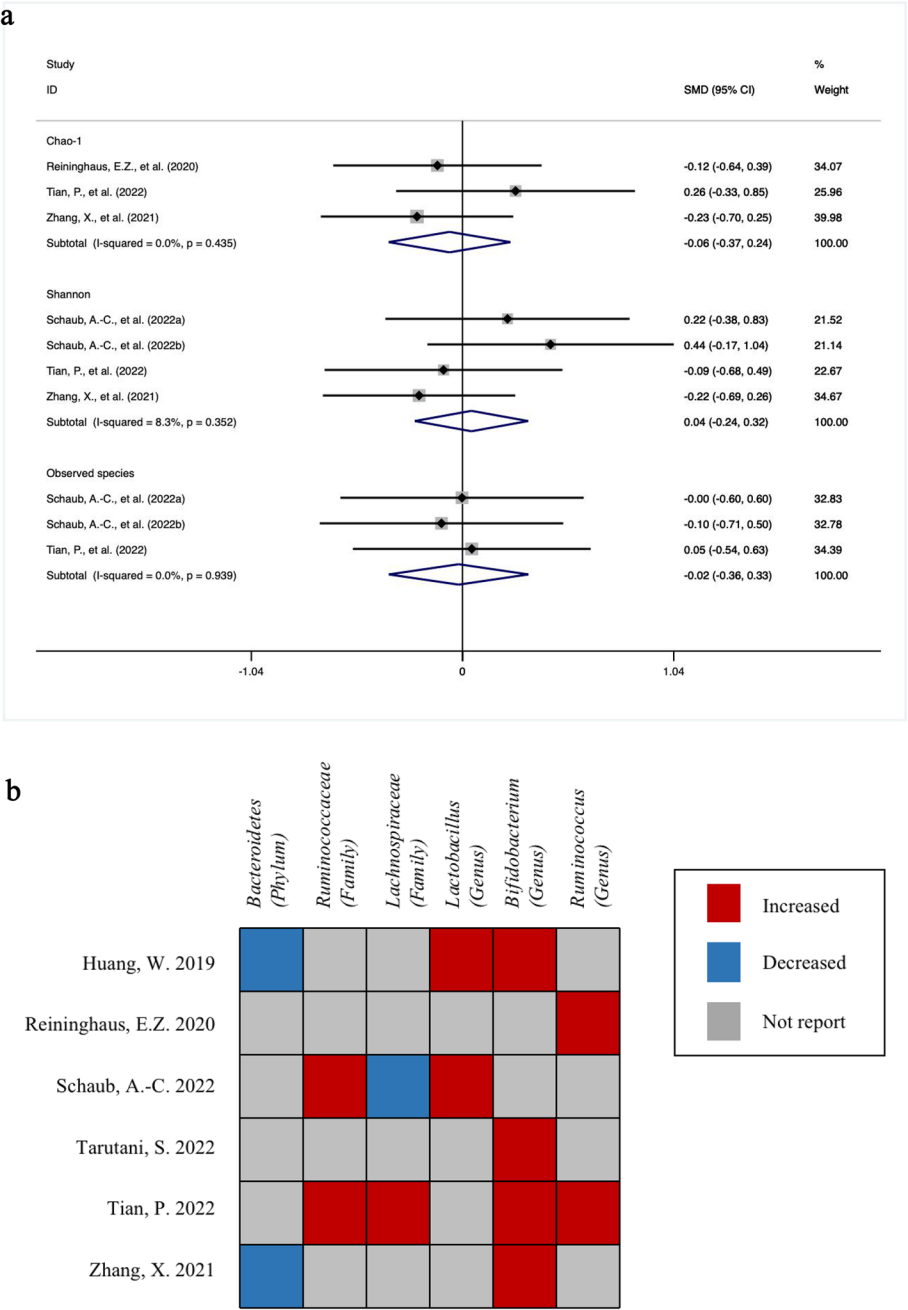
The change of α diversity and the abundance of microbiome taxa. **a** the change of Chao-1, Shannon and observed species indices in the intervention group compared with the placebo. **b** the change of gut microbiome abundance after the intervention at phylum, family and genus levels

Four studies reported α diversity measured by six indices, including richness (observed species, Chao1), evenness, and richness/evenness (Shannon, Simpson, inverse Simpson). Chao1, Shannon and observed species were most frequently used in the included studies. As shown in Fig. 5a, the pooled estimate demonstrated no significant difference in the *SMD* of Chao1, Shannon and observed species between the intervention group and placebo group (Chao-1: *SMD* = -0.06 [-0.37, 0.24], *P* = 0.676; Shannon: *SMD* = 0.04 [-0.24, 0.32], *P* = 0.758; observed species: *SMD* = -0.02 [-0.36, 0.33], *P* = 0.913). Additionally, these indices included studies with low heterogeneity (Chao-1: *I*^*2*^ = 0.0%, *P* = 0.435; Shannon: *I*^*2*^ = 8.3%, *P* = 0.352; observed species: *I*^*2*^ = 0.0%, *P* = 0.939).

Regarding β diversity, 4 studies reported inconsistent outcomes. Two of them demonstrated significant difference in β diversity between the prebiotics group and the placebo group, but the other two studies exhibited the contradictory outcomes (Supplementary Table 2).

Six studies depicted the change in gut microbiome abundance after the intervention at phylum, family and genus levels. At the phylum level, *Bacteroidetes* were decreased in two studies. *Proteobacteria* and *Actinobacteria* were increased in one study. At the family level, two studies reported changes in *Ruminococcaceae* and *Lachnospiraceae.* The former was observed to grow, and the latter presented controversial outcomes. Six studies found an elevated abundance of *Lactobacillus*, *Bifidobacterium* and *Ruminococcus* at the genus level (Fig. 5b).

### Effect of prebiotics, probiotics or synbiotics on biomedical indicators related to depression

Meta-analysis was used to evaluate the changes in IL-1β (*P* = 0.965), IL-6 (*P* = 0.178) and TNF-α (*P* = 0.420) after the intervention, which were not significantly different from those that received placebo (Supplementary Figure 1). Since the data of BDNF, cortisol and other depression-related biomarkers were not sufficient to perform a valid meta-analysis, the results from each included study were summarized here. Two studies reported increased levels of BDNF, and one study showed no significant alteration [31, 32, 36]. Three studies measured the change in serum cortisol, and no significant difference was observed when comparing interventional groups with placebo groups [33, 37, 40]. In addition, increased norepinephrine (NE) levels and decreased 5-hydroxytryptamine (5-HT) levels after using probiotics combined with electroacupuncture were reported by one study [32].

### Quality of included studies and risk of bias assessment

The quality of evidence was evaluated by GRADE criteria and the primary outcome of the included studies was high certainty (Supplementary Table 3). The risk of bias was assessed using Cochrane Collaboration’s Risk of Bias Tool 2. A total of 46.2% of studies had a low-risk overall bias, and 23.1% of studies showed high risks in general bias, leaving 30.8% of studies with some concerns for overall bias (Supplementary Figure 2). The adoption of per-protocol instead of intent-to-treat analysis and the problems in the randomization process led to the increased risk of bias.

### Sensitivity analysis and publication bias

Publication bias was tested both qualitatively and quantitatively. The funnel plot appeared to have a fair amount of symmetry, which indicated no evidence of publication bias (Supplementary Figure 3). Begg’s test (*P* = 0.236 > 0.05) and Egger’s test (*P* = 0.774 > 0.05) also revealed no publication bias. In the sensitivity analysis, no single study significantly impacted the overall results (Supplementary Figure 4).

## Discussions

In this meta-analysis, the overall effects of probiotics, prebiotics and synbiotics on depressive symptoms were significantly superior to those of placebo. Subgroup and meta-regression analyses explored the relative factors that correlated with therapeutic efficacy and the outcomes were further discussed here. The results remained significant regardless of different assessment time points and scales, which manifested the robustness of their effectiveness. Additionally, these microbiota management agents were effective for patients with both mild and moderate depression. Since antidepressant drugs had greater efficacy in severe depression, the microbiota management agents could be beneficial alternatives for those with mild-to-moderate depression. Meanwhile, Chahwan et al. showed that patients in the mild-to-moderate severity range reported the lower cognitive vulnerability to depression following probiotic intervention [42].

As for comparisons between prebiotics, probiotics and synbiotics, most studies utilized probiotics as the intervention. Subgroup analysis presented significant pooled effects of agents containing probiotics (i.e., probiotics and synbiotics) in improving depressive symptoms. However, there was only one article on synbiotics that showed significant antidepressant effects. In addition, three trials of prebiotics demonstrated no significant difference between prebiotics and placebo [30, 31, 33, 34, 39]. Liu et al. conducted a meta-analysis on prebiotics and probiotics for depression, which was in line with our results. They observed no difference between prebiotics and control conditions in reducing depressive symptom scores, while probiotics exerted significant antidepressant effects [18]. Given the insufficient number of studies on prebiotics and synbiotics, it might be premature to conclude their clinical efficacy in alleviating depressive symptoms. Therefore, further clinical trials to reveal the potential effectiveness of prebiotics and synbiotics are strongly suggested in the future. Regarding probiotics, *Lactobacillus casei (L. casei), Lactobacillus acidophilus (L. acidophilus)* and *Bifidobacterium (e.g., B. longum, B. bifidum, B. breve)* are common strains contained in probiotic capsules, working as beneficial microbial flora. *L. casei* is typically applied to treat gastrointestinal diseases, with relatively less evidence in treating depression. Animal studies have shown that it can improve depression-like behaviours in rats by reversing changes in the expression of brain-derived neurotrophic factor (BDNF) and its receptor induced by chronic stress [43]. *L. acidophilus* has been observed to mitigate lactose intolerance, enhance host immune function and inhibit the progression of cardiovascular disease, since it was initially isolated in 1990 [44]. The *Bifidobacterium* genus is a promising candidate for the treatment of psychiatric disorders. *B. breve* was found to exert antidepressant-like effects through various mechanisms, such as the deregulation of hyperactive hypothalamic–pituitary–adrenal (HPA) axis [45]. *B. longum* was shown to reduce limbic reactivity to weaken responses to negative emotional stimulation in the brain [46]. In addition, new strains of probiotics are continuously being discovered and applied, which provides diverse choices to determine suitable probiotics for depression treatment. Besides, prebiotics, probiotics and synbiotics served as add-on treatments in most studies. Whether they could replace antidepressant drugs as a first-line treatment still lacks supportive evidence. In addition, the treatment duration of prebiotics, probiotics and synbiotics did not influence their efficacy based on our subgroup outcomes. The results appeared to be nonsignificant in the subgroups with the interventions of more than 8 weeks, mainly because those studies applied prebiotics.

Sex was another factor that significantly influenced the efficacy of the intervention, as demonstrated by the subgroup and meta-regression results. Several human studies have found an influence of sex on gastrointestinal microbial composition, either in healthy individuals or depressive patients. Healthy females were reported to have a higher abundance of the *Bacteroides* genus than males. In contrast, the gut microbiota in males contained a higher abundance of *Escherichia* and *Veillonella* genera than in females [47, 48]. Concerning microbiota patterns in patients with depression, in comparison with the sex-matched healthy cohort, drug-free females with a first depressive episode had a higher abundance of *Actinobacteria*, while males had a lower abundance of *Bacteroides* [49]. Estrogen might play a role in the sex difference in microbial composition. Several studies have revealed bilateral ovariectomy-induced gut microbial dysbiosis in mice [50, 51]. Based on sex differences in gut microbiota, biological sex might also impact the response of depressive patients to treatments targeting gut microbiota such as prebiotics, probiotics and synbiotics [52]. In the study conducted by Karunasena et al., mice were fed a probiotic (i.e., *Lactobacillus animalis*). The results showed that the *Staphylococcus* and *Roseburia* genera were consistently overrepresented in females compared to males, which indicated that host response to probiotics was sex sensitive [53]. Apart from sex-specific microbial changes after the intervention, the immune system also reacted differently between males and females. Mu et al. found that *Lactobacillus* treatment was anti-inflammatory by reducing IL-6 and increasing IL-10 production in the gut in female and castrated male mice but not in intact males [54]. Sex-dependent changes in gut microbiota and immunity could explain the sex-dependent improvements in depressive symptoms after the treatment with probiotics, prebiotics, and synbiotics. Since most studies included female participants and few focused on the sex difference in efficacy, future research should explore the interactions between sex, depression and gut microbiota.

The antidepressant effects of probiotics, prebiotics and synbiotics could be explained by multiple mechanisms correlated with our secondary outcomes. The first and most direct role of prebiotics, probiotics or synbiotics was interaction with gut microbiota and their ecosystem. The animal trials of Abildgaard et al. observed a difference in the internal microbiota composition between responders and non-responders to probiotics regarding depressive-like behavior. They found that fecal abundance of relative genera, particularly the *Lactobacillus* genus, was higher in responders than in non-responders [55]. In other words, probiotics exert antidepressant effects by altering the internal microbiota. Another preclinical study illustrated that the modulation of gut microbiota and intestinal mucosa function via fecal microbiota transplantation probably contributed to alleviating depressive-like behavior [56]. In addition, the use of antibiotics leads to predisposition to depression by changing the gut microbiota. Ido Lurie et al., used a large population-based medical record database from the UK to conduct 3 nested case–control studies, and found that the use of antibiotics was associated with an increased risk for depression and recurrent antibiotic exposure could further increase that risk [57]. In another study, antibiotic mixtures were used to induce depression mouse model and caused changes in depression-related biomarkers. The species of intestinal microbiota in antibiotic-induced depression mice also underwent significant alterations, such as increased *Bacteroides* and *Klebsiella* [58]. Probiotics, in contrast, were proved to be capable of recovering the dysregulation of gut microbiota. Our quantitative analyses of the change in α diversity after the intervention appeared nonsignificant compared with the placebo, while qualitative analyses of β diversity showed inconsistent results. Insufficient sample sizes could explain these outcomes in the analyses and a specific number of microbial species in each included study. Regarding the abundance alteration of several microbial taxa, previous studies demonstrated that the *Bifidobacterium* genus was decreased in patients with MDD compared to controls [59, 60]. Our analysis revealed that the *Bifidobacterium* genus was increased after the intervention along with improvements in depressive symptoms. Indeed, more research is necessary to elucidate the explicit association between gut microbiota and depression treatment such as prebiotics, probiotics and synbiotics. The second mechanism is referred to as immune modulation. MDD patients were found to have increased proinflammatory cytokines and acute phase proteins such as IL-6, TNF, and C-reactive protein in the blood [61]. Meanwhile, probiotics and prebiotics have been shown by several studies to exhibit anti-inflammatory effects [16, 62]. Probiotics could increase anti-inflammatory cytokine levels, such as TNF, while prebiotics were discovered to reduce type 2 T helper responses [13]. However, different effects might be observed depending on the different prebiotics or probiotics used. Some have a pro-inflammatory effect, whereas others are more anti-inflammatory [63]. This partially accounted for the nonsignificant pooled results when comparing inflammatory indicators between the intervention and placebo groups. The small number of studies included in the analyses was another critical reason. These microbiota management agents might also exert their effects on the brain through other avenues, including the vagus nerve, HPA axis, microbial metabolites and neurotransmitter serotonin [64]. Therefore, future studies were suggested to examine these mechanisms to extend the current understanding of microbiota management agents.

## Strengths and limitations

Since the microbiota-gut-brain axis has great interest from clinical doctors and scientific researchers, several meta-analyses have been conducted to conclude the effects of probiotics or prebiotics in improving depressive symptoms [18, 19, 65, 66]. Compared to previous studies, this meta-analysis has the following advantages. In terms of included participants, previous studies included healthy populations and comorbid depression patients (e.g., with comorbidity of IBS), which caused high heterogeneity within populations. Depression patients, therefore, could not acquire helpful guidance on whether they should choose microbiota management agents. This study only included depression patients without major comorbidities to make a valid and generalized conclusion for patients with depression. Second, no previous meta-analysis has comprehensively examined and compared the effects of prebiotics, probiotics, and synbiotics on depression. Alli et al. conducted a systematic review on the benefits of prebiotics, probiotics and synbiotics to depressive patients, but it did not include a quantitative meta-analysis, which was exhibited in our study [20]. With regard to the outcomes, not only were the improvements in depressive symptoms evaluated, but gut microbiota indices and inflammatory indicators were also compared between the intervention and placebo groups. Finally, the data in our study were very recent, as 10 of 13 included studies were published between 2019 and 2022 and were not included in the previous meta-analyses.

Nevertheless, certain limitations existed in the meta-analysis. First, the included studies and sample sizes were relatively small, especially those that utilized prebiotics or synbiotics. Therefore, more studies are warranted to support the antidepressant effects of prebiotics and synbiotics. Second, since the two included studies contained three parallel groups, namely prebiotics, probiotics and placebo, we evenly divided the number of patients in the placebo group to accomplish the pooled analysis. However, it only partially overcame the unit-of-analysis error. Third, different microbiomes, intervention durations, outcome measurements and assessment time points in different studies could interfere with the primary result (i.e., reductions in depressive symptom scores). Thus, we conducted various subgroup and meta-regression analyses to further describe the primary outcome. Fourth, prebiotics, probiotics and synbiotics were applied as add-on treatments in most studies with a paucity of identification of their isolated effects. Moreover, antidepressants were reported to have antimicrobial effects, which probably influence the efficacy of microbiota management agents [67]. Fifth, the included studies recruited few males and adolescents, but sex and age might impact the effects of probiotics, prebiotics and synbiotics. For sex, our subgroup analysis and meta-regression demonstrated its influence. In terms of age, several studies have reported that microbiome composition and abundance differed across the lifespan [68, 69]. Therefore, a balanced sex and age distribution is expected in future clinical trials.

## Conclusions

In conclusion, agents that manipulate gut microbiota might become a novel approach to treat patients with mild-to-moderate depression. Significant antidepressant effects of probiotics were observed, whereas the efficacy of prebiotics and synbiotics on depression requires more evidence to confirm. In addition, biological sex was a vital factor that influenced patients’ responses to the treatment, and research and real-world practice could focus more on this point. Finally, inconsistent outcomes and insufficient data on the changes in gut microbiota and inflammatory indicators warrant future studies to investigate more on the mechanisms of prebiotics, probiotics and synbiotics.

## Supplementary Information

**Additional file 1: Supplementary Table 1.** Search Strategy. **Supplementary Table 2.** Summary of β diversity in the included studies. **Supplementary Table 3.** GRADE summary of studies. **Supplementary Figure 1.** The change of inflammatory indicators including IL-1β, IL-6 and TNF-α. **Supplementary Figure 2.** Risk of bias graph assessed by Cochrane Collaboration’s Risk of bias Tool 2. (a) displayed the domain and overall judgements of risk of bias study-by-study, and (b) showed the percentage of risk of bias assessments at each level of risk of bias per domain. **Supplementary Figure 3.** Funnel plot that examined publication bias. **Supplementary Figure 4.** A sensitivity analysis that tested the robustness of the outcomes.

## Data Availability

The data used for this meta-analysis are publicly available in the research studies. The full dataset can be requested from the corresponding author on reasonable request.

## Abbreviations

BDI: Beck Depression Inventory
BMI: Body mass index
CNS: The central nervous system
EPDS: Edinburgh Postnatal Depression Scale
GRADE: Grading of Recommendation, Assessment, Development, and Evaluation
HDRS: Hamilton Depression Rating Scale
IBS: Irritable bowel syndrome
MADRS: Montgomery Asberg Depression Rating Scale
MDD: Major depressive disorder
MeSH: Medical Subject Heading
PRISMA: Preferred Reporting Items for Systematic Reviews and Meta-analyses
RCTs: Randomized controlled trials
SD: Standard deviation
SMD: Standardized mean difference
WHO: World Health Organization
