# Supplementary material for: Effect of prebiotics, probiotics, synbiotics on depression: results from a meta-analysis
Source: BMC Psychiatry. 2023 Jun 29;23:477. doi: 10.1186/s12888-023-04963-x (PMC10308754; doi:10.1186/s12888-023-04963-x)
Supplement: Supplementary file 1 — Additional file 1: Supplementary Table 1. Search Strategy. Supplementary Table 2. Summary of β diversity in the included studies. Supplementary Table 3. GRADE summary of studies. Supplementary Figure 1. The change of inflammatory indicators including IL-1β, IL-6 and TNF-α. Supplementary Figure 2. Risk of bias graph assessed by Cochrane Collaboration’s Risk of bias Tool 2. (a) displayed the domain and overall judgements of risk of bias study-by-study, and (b) showed the percentage of risk of bias assessments at each level of risk of bias per domain. Supplementary Figure 3. Funnel plot that examined publication bias. Supplementary Figure 4. A sensitivity analysis that tested the robustness of the outcomes. [file 12888_2023_4963_MOESM1_ESM.docx]

**Supplementary Table 1. Search Strategy**

| **Databases** | **Search terms** | **Number of records** |
| --- | --- | --- |
| **PubMed** | #1 "Depression"[Mesh]  #2 "Depressive Disorder"[Mesh]  #3 ((((((((((((((((((((((("depressive state") OR ("depressive symptoms")) OR ("depressive symptom")) OR ("emotional depression")) OR ("depressive disorders")) OR ("depressive disease")) OR ("depressive episode")) OR ("depressive illness")) OR ("depressive personality disorder")) OR ("depressive neuroses")) OR ("depressive neurosis")) OR ("endogenous depression")) OR ("endogenous depressions")) OR ("depressive syndrome")) OR ("depressive syndromes")) OR ("neurotic depression")) OR ("neurotic depressions")) OR ("melancholia")) OR ("melancholias")) OR ("unipolar depression")) OR ("unipolar depressions")) OR ("central depression")) OR ("clinical depression")) OR ("mental depression")  #4 #1 or #2 or #3 ----276,616 results  #5 ((("Prebiotics"[Mesh]) OR ("Probiotics"[Mesh])) OR ("Synbiotics"[Mesh])) OR ((("Prebiotic") OR ("Probiotic")) OR ("Synbiotic"))  #6 ((("Gastrointestinal Microbiome"[Mesh]) OR ((((((((((((((((((((((((((((((((((((((((((((((("Gastrointestinal Microbiomes") OR ("Gastrointestinal Flora")) OR ("Gastrointestinal Microbiota")) OR ("Gastrointestinal Microbiotas")) OR ("Gastrointestinal Microbial Community")) OR ("Gastrointestinal Microbial Communities")) OR ("Gastrointestinal Microflora")) OR ("gastrointestinal canal flora")) OR ("gastrointestinal tract flora")) OR ("gastrointestine tract flora")) OR ("gastrointestine flora")) OR ("Gut Microbiome")) OR ("Gut Microbiomes")) OR ("Gut Microflora")) OR ("Gut Microbiota")) OR ("Gut Microbiotas")) OR ("Gut Flora")) OR ("gut bacteria")) OR ("Gastric Microbiome")) OR ("Gastric Microbiomes")) OR ("Intestinal Microbiome")) OR ("Intestinal Microbiomes")) OR ("Intestinal Microbiota")) OR ("Intestinal Microbiotas")) OR ("Intestinal Microflora")) OR ("intestinal microorganism")) OR ("Intestinal Flora")) OR ("intestinal tract flora")) OR ("intestinal microbe")) OR ("intestinal microbes")) OR ("intestinal bacterial flora")) OR ("intestinal bacteria")) OR ("intestinal bacterium")) OR ("intestinal canal flora")) OR ("intestine bacteria change")) OR ("intestine bacterium")) OR ("intestine microbial flora")) OR ("intestine microflora")) OR ("Enteric Bacteria")) OR ("enteric flora")) OR ("enteric microbiota")) OR ("alimentary canal flora")) OR ("alimentary tract flora")) OR ("bowel flora")) OR ("bowel microbiota")) OR ("digestive canal flora")) OR ("digestive tract flora"))) OR ("intestinal virome")) OR ("gut virome")  #7 #4 AND #5 AND #6 | **154 results** |
| **Cochrane Library** | #1 MeSH descriptor: [Depression] explode all trees  #2 (‘depression’) OR (‘depressive state’) OR (‘depressive symptoms’) OR (‘depressive symptom’) OR (‘emotional depression’) (Word variations have been searched)  #3 (‘depressive disorders’) OR (‘depressive disease’) OR (‘depressive episode’) OR (‘depressive illness’) OR (‘depressive personality disorder’) (Word variations have been searched)  #4 (‘depressive neuroses’) OR (‘depressive neurosis’) OR (‘endogenous depression’) OR (‘endogenous depressions’) OR (‘depressive syndrome’) (Word variations have been searched)  #5 (‘depressive syndromes’) OR (‘dneurotic depression’) OR (‘neurotic depressions’) OR (‘melancholia’) OR (‘melancholias’) (Word variations have been searched)  #6 (‘unipolar depression’) OR (‘unipolar depressions’) OR (‘central depression’) OR (‘clinical depression’) OR (‘mental depression’) (Word variations have been searched)  #7 #1 or #2 or #3 or #4 or #5 or #6  #8 MeSH descriptor: [Prebiotics] explode all trees  #9 MeSH descriptor: [Probiotics] explode all trees  #10 MeSH descriptor: [Synbiotics] explode all trees  #11 (‘Prebiotics’) OR (‘Probiotics’) OR (‘Synbiotics’) OR (‘Prebiotic’) OR (‘Probiotic’) (Word variations have been searched)  #12 (‘Synbiotic’) (Word variations have been searched)  #13 #8 or #9 or #10 or #11 or #12  #14 MeSH descriptor: [Gastrointestinal Microbiome] explode all trees  #15 (‘Gastrointestinal Microbiomes’) OR ('Gastrointestinal Flora') OR ('Gastrointestinal Microbiota') OR ('Gastrointestinal Microbiotas') OR ('Gastrointestinal Microbial Community') (Word variations have been searched)  #16 (‘Gastrointestinal Microbial Communities’) OR ('Gastrointestinal Microflora') OR ('gastrointestinal canal flora') OR ('gastrointestinal tract flora') OR ('gastrointestine tract flora') (Word variations have been searched)  #17 (‘gastrointestine flora’) OR ('Gut Microbiome') OR ('Gut Microbiomes') OR ('Gut Microflora') OR ('Gut Microbiota') (Word variations have been searched)  #18 (‘Gut Microbiotas’) OR ('Gut Flora') OR ('gut bacteria') OR ('Gastric Microbiome') OR ('Gastric Microbiomes') (Word variations have been searched)  #19 (‘Intestinal Microbiome’) OR ('Intestinal Microbiomes') OR ('Intestinal Microbiota') OR ('Intestinal Microbiotas') OR ('Intestinal Microflora') (Word variations have been searched)  #20 (‘Iintestinal microorganism’) OR ('IIntestinal Flora') OR ('intestinal tract flora') OR ('intestinal microbe') OR ('intestinal microbes') (Word variations have been searched)  #21 (‘intestinal bacterial flora’) OR ('intestinal bacteria') OR ('intestinal bacterium') OR ('intestinal canal flora') OR ('intestine bacteria change') (Word variations have been searched)  #22 (‘intestine bacterium’) OR ('intestine microbial flora') OR ('intestine microflora') OR ('Enteric Bacteria') OR ('enteric flora') (Word variations have been searched)  #23 (‘enteric microbiota’) OR ('alimentary canal flora') OR ('alimentary tract flora') OR ('bowel flora') OR ('bowel microbiota') (Word variations have been searched)  #24 (‘digestive canal flora’) OR ('digestive tract flora') OR (‘intestinal virome’) OR ('gut virome') (Word variations have been searched)  #25 #14 or #15 or #16 or #17 or #18 or #19 or #20 or #21 or #22 or #23 or #24  #26 #7 and #13 and #25 | **158 results** |
| **EMBASE** | #1. 'depression'/exp  #2. 'depression' OR 'depressive state' OR 'depressive symptoms' OR 'depressive symptom' OR 'emotional depression' OR 'depressive disorders' OR 'depressive disease' OR 'depressive episode' OR 'depressive illness' OR 'depressive personality disorder' OR 'depressive neuroses' OR 'depressive neurosis' OR 'endogenous depression' OR 'endogenous depressions' OR 'depressive syndrome' OR 'depressive syndromes' OR 'neurotic depression' OR 'neurotic depressions' OR 'melancholia' OR 'melancholias' OR 'unipolar depression' OR 'unipolar depressions' OR 'central depression' OR 'clinical depression' OR 'mental depression'  #3. #1 OR #2  #4. 'prebiotic agent'/exp  #5. 'probiotic agent'/exp  #6. 'synbiotic agent'/exp  #7. 'prebiotics'/exp OR 'prebiotics' OR 'probiotics' OR 'synbiotics' OR 'prebiotic' OR 'probiotic' OR 'synbiotic' OR 'prebiotic agent' OR 'probiotic agent' OR 'synbiotic agent'  #8. #4 OR #5 OR #6 OR #7  #9. 'intestine flora'/exp  #10. 'gastrointestinal microbiomes' OR 'gastrointestinal flora' OR 'gastrointestinal microbiota' OR 'gastrointestinal microbiotas' OR 'gastrointestinal microbial community' OR 'gastrointestinal microbial communities' OR 'gastrointestinal microflora' OR 'gastrointestinal canal flora' OR 'gastrointestinal tract flora' OR 'gastrointestine tract flora' OR 'gastrointestine flora' OR 'gut microbiome' OR 'gut microbiomes' OR 'gut microflora' OR 'gut microbiota' OR 'gut microbiotas' OR 'gut flora' OR 'gut bacteria'  #11. 'gastric microbiome' OR 'gastric microbiomes' OR 'intestinal microbiome' OR 'intestinal microbiomes' OR 'intestinal microbiota' OR 'intestinal microbiotas' OR 'intestinal microflora' OR 'intestinal microorganism' OR 'intestinal flora' OR 'intestinal tract flora' OR 'gastrointestine flora' OR 'intestinal microbe' OR 'intestinal microbes' OR 'intestinal bacterial flora' OR 'intestinal bacteria' OR 'intestinal bacterium' OR 'intestinal canal flora' OR 'intestine bacteria change' OR 'intestine bacterium' OR 'intestine microbial flora' OR 'intestine microflora'  #12. 'enteric bacteria' OR 'enteric flora' OR 'alimentary canal flora' OR 'alimentary tract flora' OR 'bowel flora' OR 'bowel microbiota' OR 'digestive canal flora' OR 'digestive tract flora' OR 'iintestinal virome' OR 'gut virome'  #13. #9 OR #10 OR #11 OR #12  #14. #3 AND #8 AND #13 | **838 results** |
| **Medline** | S1 TX ‘depression’ OR TX Depressive Disorder OR TX depressive state OR TX depressive symptoms OR TX depressive symptom OR TX emotional depression OR TX depressive disorders OR TX depressive disease OR depressive episode OR depressive illness OR TX depressive personality disorder OR TX depressive neuroses OR TX depressive neuroses OR TX endogenous depression OR TX endogenous depressions OR TX depressive syndrome OR TX depressive syndromes OR TX neurotic depression OR TX neurotic depressions OR TX melancholia OR TX melancholias OR TX unipolar depression OR TX unipolar depressions OR TX central depression OR TX depressive episode OR TX depressive illness OR TX clinical depression OR TX mental depression  S2 TX Prebiotics OR TX probiotics OR TX synbiotics OR TX prebiotic OR TX probiotic OR TX synbiotic OR TX prebiotic agent OR TX probiotic agent OR TX synbiotic agent  S3 TX gastrointestinal microbiome OR TX Gastrointestinal Microbiomes OR TX Gastrointestinal Flora OR TX gastrointestinal microbiota OR TX Gastrointestinal Microbiotas OR TX Gastrointestinal Microbial Community OR TX Gastrointestinal Microbial Communities OR TX Gastrointestinal Microflora OR TX gastrointestinal canal flora OR TX gastrointestinal tract flora OR TX gastrointestine tract flora OR TX gastrointestine flora OR TX gut microbiome OR TX Gut Microbiomes OR TX gut microflora OR TX gut microbiota OR TX gut microbiomes OR TX gut flora OR TX gut bacteria OR TX Gastric Microbiome OR TX Gastric Microbiomes OR TX intestinal microbiome OR TX intestinal microbiome OR TX intestinal microbiota OR TX Intestinal Microbiotas OR TX Intestinal Microflora OR TX intestinal microorganism OR TX intestinal flora OR TX intestinal tract flora OR TX intestinal microbe OR TX intestinal microbes OR TX intestinal bacterial flora OR TX intestinal bacteria OR TX intestinal bacterium OR TX intestinal canal flora OR TX intestine bacteria change OR TX intestine bacterium OR TX intestine microbial flora OR TX Enteric Bacteria OR TX enteric flora OR TX enteric microbiota OR TX alimentary canal flora OR TX alimentary tract flora OR TX bowel flora OR TX bowel microbiota OR TX digestive canal flora OR TX digestive tract flora OR TX intestinal virome  S4 S1 and S2 and S3 | **898 results** |
| **Web of Science** | #1 depression or depressive state or depressive symptoms or depressive symptom or emotional depression or Depressive Disorder or depressive disorders or depressive disease or depressive episode or depressive illness or depressive personality disorder or depressive neuroses or depressive neurosis or endogenous depression or endogenous depressions or depressive syndrome or depressive syndromes or neurotic depression or neurotic depressions or melancholia or melancholias or unipolar depression or unipolar depressions or central depression or clinical depression or mental depression  #2 Gastrointestinal Microbiome or intestine flora or Gastrointestinal Microbiomes or Gastrointestinal Flora or Gastrointestinal Microbiota or Gastrointestinal Microbiotas or Gastrointestinal Microbial Community or Gastrointestinal Microbial Communities or Gastrointestinal Microflora or gastrointestinal canal flora or gastrointestinal tract flora or gastrointestine tract flora or gastrointestine flora or Gut Microbiome or Gut Microbiomes or Gut Microflora or Gut Microbiota or Gut Microbiotas or Gut Flora or gut bacteria or Gastric Microbiome or Gastric Microbiomes  #3 Intestinal Microbiome or Intestinal Microbiomes or Intestinal Microbiota or Intestinal Microbiotas or Intestinal Microflora or intestinal microorganism or Intestinal Flora or intestinal tract flora or intestinal microbe or intestinal microbes or intestinal bacterial flora or intestinal bacteria or intestinal bacterium or intestinal canal flora or intestine bacteria change or intestine bacterium or intestine microbial flora or intestine microflora or Enteric Bacteria or enteric flora or enteric microbiota or alimentary canal flora or alimentary tract flora or bowel flora or bowel microbiota or digestive canal flora or digestive tract flora or intestinal virome or gut virome  #4 Prebiotics or Probiotics or Synbiotics or prebiotic agent or probiotic agent or synbiotic agent or Prebiotic or Probiotic or Synbiotic  #5 #2 OR #3  #6 #5 AND #4 AND #1 | **787 results** |
| **psycINFO** | S1 TX ‘depression’ OR TX Depressive Disorder OR TX depressive state OR TX depressive symptoms OR TX depressive symptom OR TX emotional depression OR TX depressive disorders OR TX depressive disease OR depressive episode OR depressive illness OR TX depressive personality disorder OR TX depressive neuroses OR TX depressive neuroses OR TX endogenous depression OR TX endogenous depressions OR TX depressive syndrome OR TX depressive syndromes OR TX neurotic depression OR TX neurotic depressions OR TX melancholia OR TX melancholias OR TX unipolar depression OR TX unipolar depressions OR TX central depression OR TX depressive episode OR TX depressive illness OR TX clinical depression OR TX mental depression  S2 TX Prebiotics OR TX probiotics OR TX synbiotics OR TX prebiotic OR TX probiotic OR TX synbiotic OR TX prebiotic agent OR TX probiotic agent OR TX synbiotic agent  S3 TX gastrointestinal microbiome OR TX Gastrointestinal Microbiomes OR TX Gastrointestinal Flora OR TX gastrointestinal microbiota OR TX Gastrointestinal Microbiotas OR TX Gastrointestinal Microbial Community OR TX Gastrointestinal Microbial Communities OR TX Gastrointestinal Microflora OR TX gastrointestinal canal flora OR TX gastrointestinal tract flora OR TX gastrointestine tract flora OR TX gastrointestine flora OR TX gut microbiome OR TX Gut Microbiomes OR TX gut microflora OR TX gut microbiota OR TX gut microbiomes OR TX gut flora OR TX gut bacteria OR TX Gastric Microbiome OR TX Gastric Microbiomes OR TX intestinal microbiome OR TX intestinal microbiome OR TX intestinal microbiota OR TX Intestinal Microbiotas OR TX Intestinal Microflora OR TX intestinal microorganism OR TX intestinal flora OR TX intestinal tract flora OR TX intestinal microbe OR TX intestinal microbes OR TX intestinal bacterial flora OR TX intestinal bacteria OR TX intestinal bacterium OR TX intestinal canal flora OR TX intestine bacteria change OR TX intestine bacterium OR TX intestine microbial flora OR TX Enteric Bacteria OR TX enteric flora OR TX enteric microbiota OR TX alimentary canal flora OR TX alimentary tract flora OR TX bowel flora OR TX bowel microbiota OR TX digestive canal flora OR TX digestive tract flora OR TX intestinal virome  S4 S1 and S2 and S3 | **128 results** |

**Supplementary Table 2. Summary of β diversity in the included studies**

| **Study** | **Methods** | **Findings of β diversity** |
| --- | --- | --- |
| Reininghaus, E.Z., et al. (2020) | PCA | There was significant difference in gut microbial β diversity between the probiotics and the placebo group. |
| Schaub, A.C., et al. (2022) | PCoA based on Bray-Curtis index | There was significant difference in gut microbial β diversity between the probiotics and the placebo group. |
|  | The stepwise confounding analysis | Subject and moisture explained 84% of the microbiome variation. |
| Tian, P., et al. (2022) | PCA | There was no significant difference in gut microbial β diversity between the probiotics and the placebo group. |
| Zhang, X., et al. (2021) | PCoA based on Bray–Curtis index | There was no significant difference in gut microbial β diversity between the probiotics and the placebo group. |

PCA: Principal component analysis, PCoA: Principal Co-ordinates Analysis

**Supplementary Table 3. GRADE summary of studies**

| Outcome | No. of studies | Risk of bias | Inconsistency | | Indirectness | Imprecision | Other considerations | Overall certainty of evidence |
| --- | --- | --- | --- | --- | --- | --- | --- | --- |
| Depressive symptom rating scores | 13 studies consisting of 22 comparisons | Not serious | | Not serious | Not serious | Not serious | None | ⨁⨁⨁⨁  High |


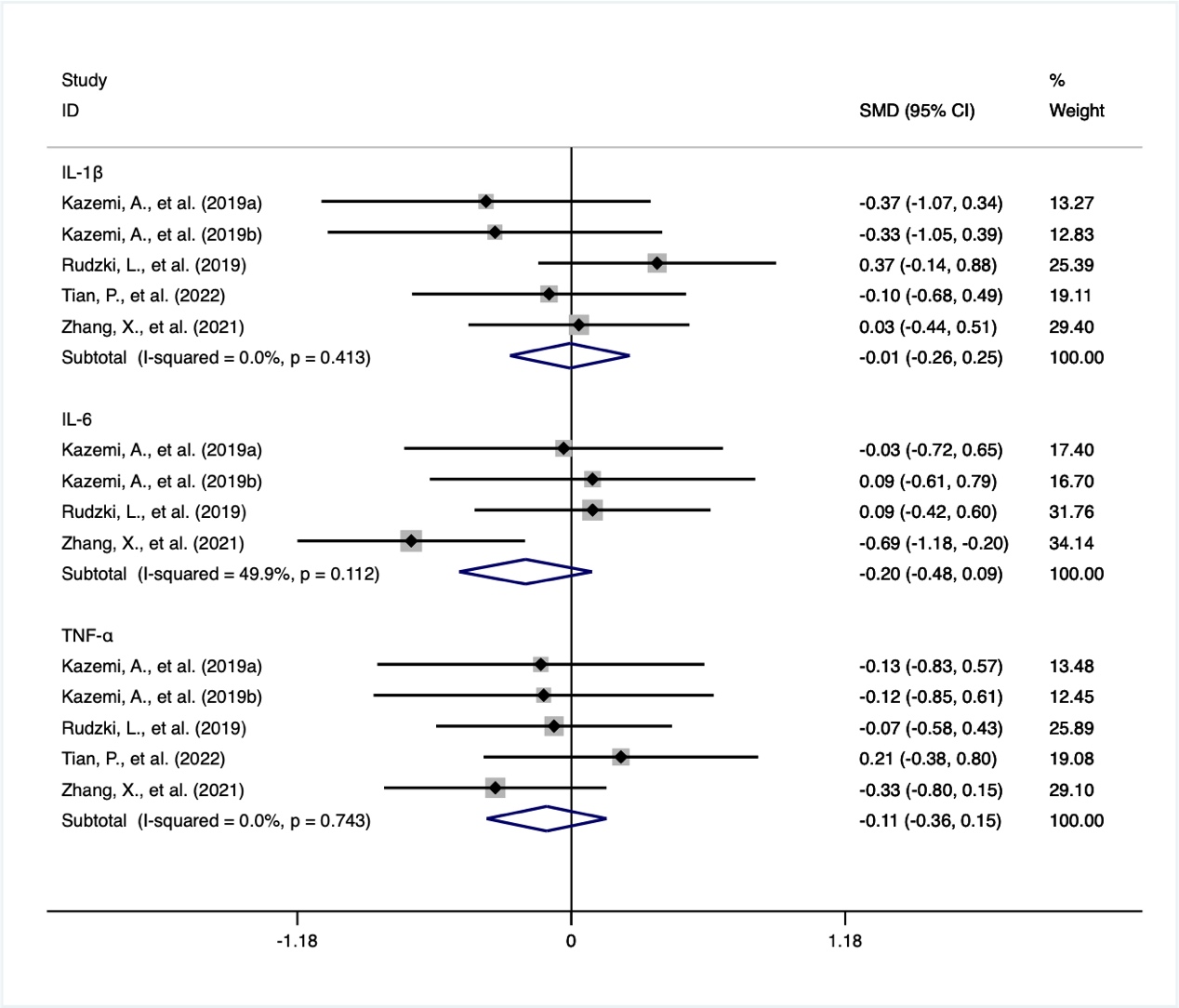


**Supplementary Figure 1.** The change of inflammatory indicators including IL-1β, IL-6 and TNF-α.


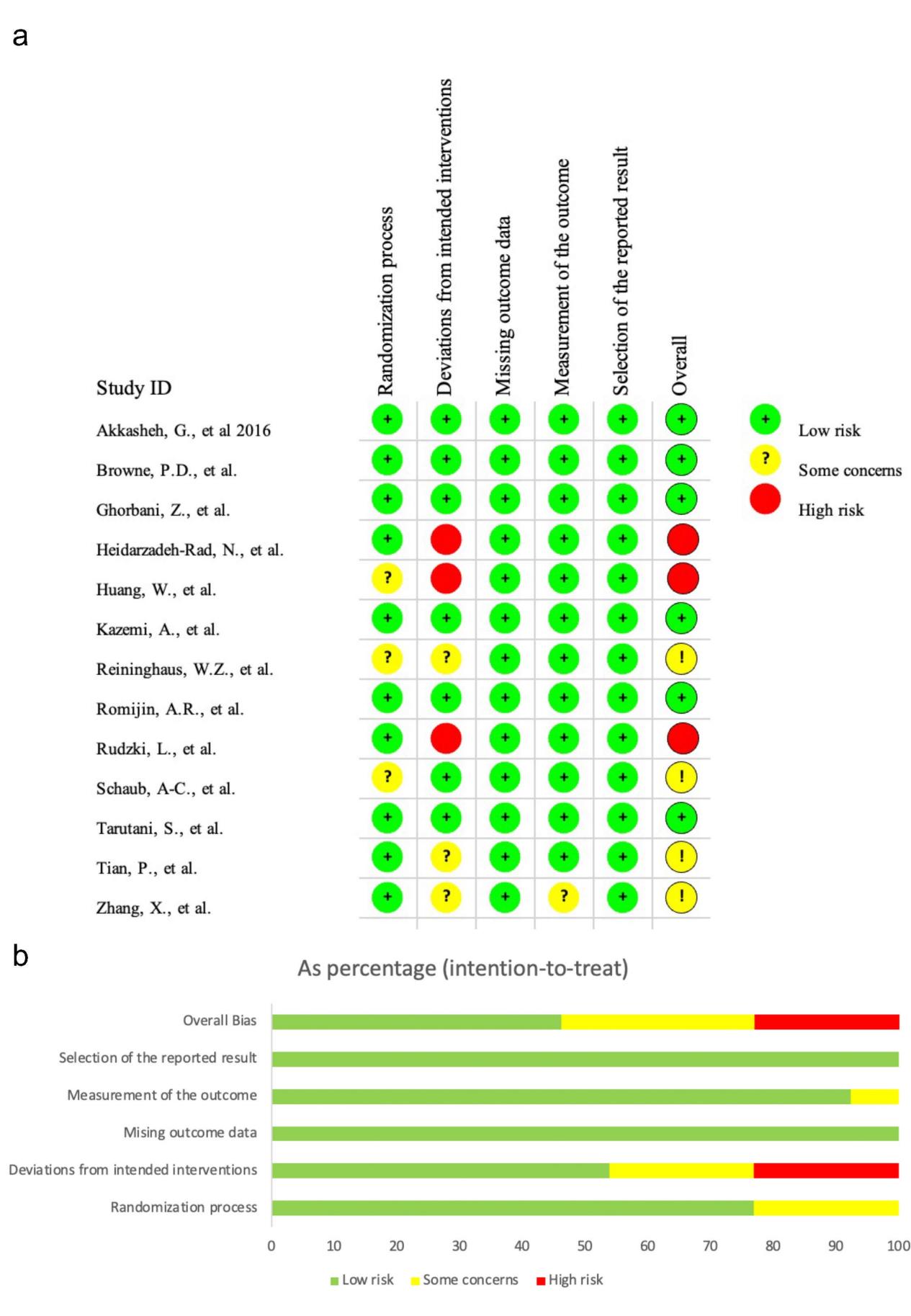


**Supplementary Figure 2.** Risk of bias graph assessed by Cochrane Collaboration’s Risk of bias Tool 2. (a) displayed the domain and overall judgements of risk of bias study-by-study, and (b) showed the percentage of risk of bias assessments at each level of risk of bias per domain.


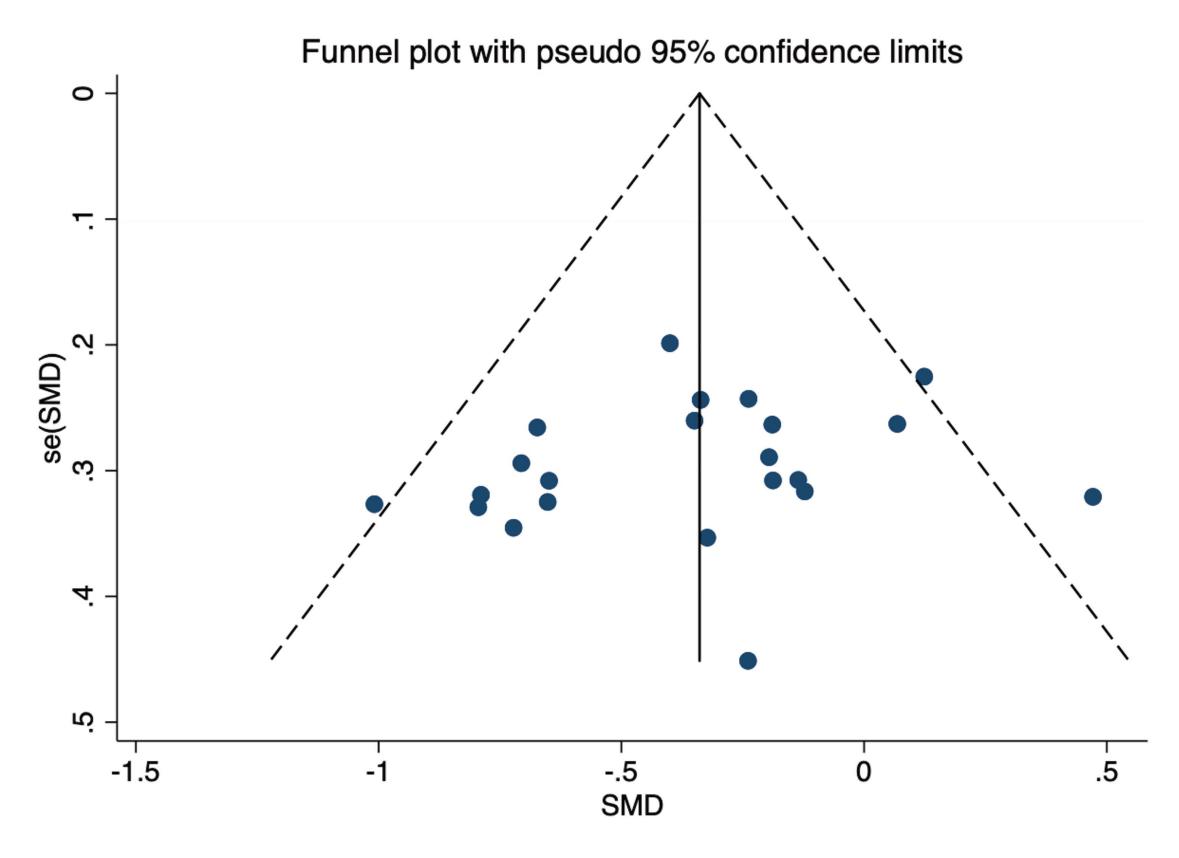


**Supplementary Figure 3.** Funnel plot that examined publication bias.


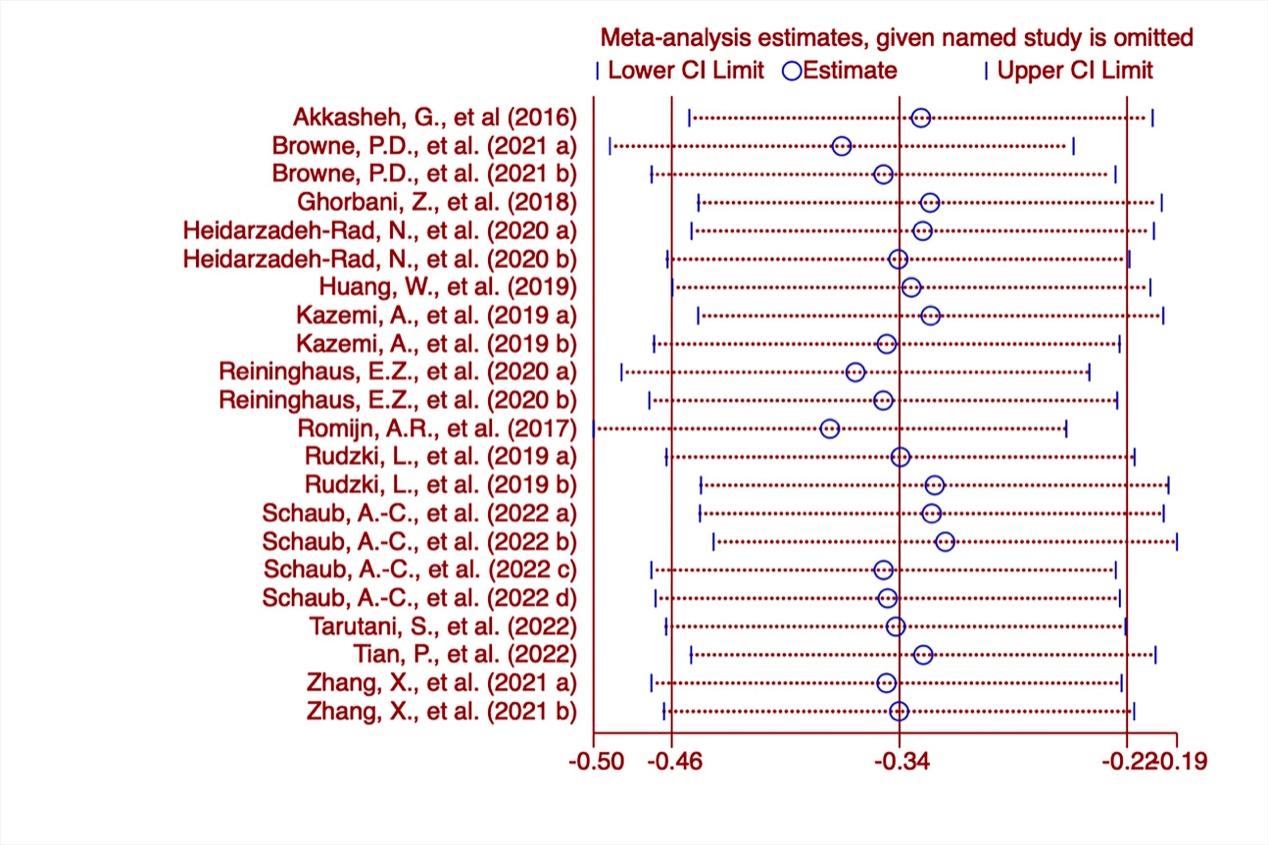


**Supplementary Figure 4.** A sensitivity analysis that tested the robustness of the outcomes.
